# Biotic acts of antibiotics

**DOI:** 10.3389/fmicb.2013.00241

**Published:** 2013-08-19

**Authors:** Rustam I. Aminov

**Affiliations:** Faculty of Medical Sciences, University of the West IndiesKingston, Jamaica

**Keywords:** tetracyclines, macrolides, β-lactam, inflammation, respiratory, cardiovascular, neuroprotection, cancer

## Abstract

Biological functions of antibiotics are not limited to killing. The most likely function of antibiotics in natural microbial ecosystems is signaling. Does this signaling function of antibiotics also extend to the eukaryotic – in particular mammalian – cells? In this review, the host modulating properties of three classes of antibiotics (macrolides, tetracyclines, and β-lactams) will be briefly discussed. Antibiotics can be effective in treatment of a broad spectrum of diseases and pathological conditions other than those of infectious etiology and, in this capacity, may find widespread applications beyond the intended antimicrobial use. This use, however, should not compromise the primary function antibiotics are used for. The biological background for this inter-kingdom signaling is also discussed.

## INTRODUCTION

We are all familiar with the use of antibiotics for treatment of infectious diseases. But antibiotics do not only kill bacteria, their original role possibly involved signaling functions (Davies et al., 2006; Linares et al., 2006; Yim et al., 2006, 2007; Martínez, 2008; Aminov, 2009; Romero et al., 2011). These functions, usually performed at lower concentrations, are different from those leading to cell death, and they are realized through different sets of molecular targets in the cell. While many aspects of this communication in the microbial world remain elusive, there is a large body of information regarding the signaling effects of low-dose antibiotics on humans and animals beyond the intended antimicrobial activities. Thus the intention of this article is to undertake an interdisciplinary coverage of and familiarize biologists with this aspect of non-antimicrobial antibiotic use in clinical research and practice. The results covered in this review have been collected in various animal models, tissue cultures, and pre-clinical and clinical trials, with little or no involvement of microbiology, and, therefore, might have escaped the attention of microbiologists. I believe this interdisciplinary coverage is highly important to close the gap in non-antimicrobial use of antimicrobials for a number of reasons. First of all, it is the specifics of this type of therapy, with the use of low-dose antibiotics for very extended periods of time measured in weeks, months, and even years. Second, while in clinical microbiology a great deal of attention is paid to the appearance of antibiotic resistance as a side effect of antibiotic therapy, this aspect has had a relatively low priority and has been largely overlooked in the low-dose long-term antibiotic treatment trials. Another aspect that may need more careful consideration in this type of therapy is the role of commensal microbiota, which is also an important player in human metabolism and physiology. Antibiotics act not only on the targets in the human body but also on the microbiota, which is the integral part of human metabolism and physiology. And as we know, the role of commensal microbiota in human health and disease is immense, affecting almost every aspect of it. Thus the antibiotic effects have to be evaluated from both sides of their activities, including the direct interaction of antibiotics with the host cells as well as indirect, through the modulation of microbiota and, correspondingly, microbial metabolites, macromolecules, and other biologically active components of microbiota that affect the host. And finally, it is intriguing to recognize how many molecular targets for antibiotics are in the human body. Is it by chance that they have such pleiotropic properties that are affecting almost every organ or system in the human body? Only three classes of antibiotics are covered in this review because of space restraints. These are the macrolides, tetracyclines, and β-lactams. For the same reason, only few most important examples for each antibiotic class and for each group of diseases are given. These are followed by a discussion of various implications of the effects and consequences of the non-antimicrobial antibiotic use.

## MACROLIDES

There are many examples of antibiotic signaling effects on the host beyond the intended antimicrobial activity. The use of macrolides for treatment of non-infectious diseases has the earliest history among other antibiotics. A considerable amount of information regarding the therapeutic potential of macrolides for non-antimicrobial use has been collected beginning from the late 1980s. Since then, a great number of animal experiments have been performed, many representatives of this class of antibiotics have gone through clinical trials, and a number of drugs in this group have been approved and are currently commonly used in clinical practice for non-antimicrobial purposes.

The use of macrolides has been especially successful in the management of various chronic respiratory diseases not only in the role of antimicrobial agents but also due to their anti-inflammatory and pro-kinetic properties. The positive effect of long-term low-dose administration of erythromycin to patients with diffuse panbronchiolitis was demonstrated by Japanese researchers more than two decades ago, thus suggesting other than antimicrobial nature of erythromycin action (Kudoh et al., 1987; Nagai et al., 1991). From this point, the use of macrolides for non-antimicrobial purposes has become one of the mainstream choices for treatment of chronic respiratory diseases.

In cystic fibrosis (CF), the main bacterium associated with the pulmonary disease is *Pseudomonas aeruginosa, *which produces biofilms resistant to antibiotic treatment within the airways (Singh et al., 2000). Although *P. aeruginosa *is naturally resistant to macrolides, these antibiotics, even at subinhibitory concentrations, can suppress quorum sensing necessary for biofilm formation (Tateda et al., 2007). This mechanism possibly contributes to the heightened sensitivity of non-susceptible *P. aeruginosa *toward a variety of anti-pseudomonal agents in biofilms when exposed to macrolides at subinhibitory concentrations (Lutz et al., 2012). In addition, low-dose macrolides display immunomodulatory properties influencing cytokine production and altering polymorphonuclear cell functions (Schultz, 2004). This prevents excessive uncontrolled inflammation and associated tissue damage. Another benefit of the macrolide use in the management of CF is the reduced chronic airway hypersecretion (Tamaoki et al., 1995).

Treatment of other respiratory diseases such as asthma may benefit from the dual action of macrolides because asthma is a result of interaction of genetic and environmental factors. The presence of *Mycoplasma pneumoniae* and *Chlamydophila pneumoniae* in asthmatics best identifies the macrolide responsive phenotype because of the antimicrobial and anti-inflammatory properties of macrolides covering the infection and genetic predisposition continuum (Good et al., 2012).

Chronic obstructive pulmonary disease (COPD) remains one of the important causes of morbidity, mortality, and health-care costs worldwide (Mannino and Buist, 2007). Although smoking is the most important risk factor for the disease, it also has a substantial genetic component (Wain et al., 2012). Pathogenesis in COPD is largely driven by dysregulated responses of the innate and adaptive immune systems to the environmental cues leading to an exaggerated inflammatory response, which results in permanent inflammation, tissue damage, and lung function decline (Holloway and Donnelly, 2013). A well-designed, randomized, 1-year trial of erythromycin, at a dose of 250 mg twice daily, has found a significant reduction in COPD exacerbations compared to the placebo group (Seemungal et al., 2008). Long-term administration of azithromycin by outpatients with severe COPD has appeared to be safe and effective, with reduced exacerbations, hospitalizations, and improved quality of life (Blasi et al., 2010). Another trial with a daily azithromycin for 1 year for prevention of exacerbations of COPD has demonstrated decreased frequency of exacerbations and improved quality of life but has caused hearing decrements in a small percentage of subjects (Albert et al., 2011). A recent review of controlled clinical studies focusing on the prevention of COPD exacerbations with long-term azithromycin, erythromycin, or clarithromycin treatment suggests that it is effective, safe, and cost-efficient (Simoens et al., 2013). Other chronic respiratory diseases may also be treated by macrolides, but better designed trials are necessary to confirm their efficacy (Suresh Babu et al., 2013).

Novel effects of macrolides on cardiovascular diseases have been discovered recently. In animal models, clarithromycin has suppressed the development of myocarditis, cardiac rejection, and myocardial ischemia (Nakajima et al., 2010; Suzuki et al., 2012). The positive effect of clarithromycin in cardiovascular diseases may be due to the alteration of inflammatory factors and matrix metalloproteinases (MMPs). MMPs as a part of the extracellular matrix participate in a number of normal physiological processes, which contribute to tissue structure, function, and remodeling, including the myocardium (Spinale et al., 2013). Both the expression and activity of MMPs are regulated by the tissue inhibitors of matrix metalloproteinases (TIMPs), and the MMPs/TIMPs balance is crucial for the normal maintenance of myocardial interstitial homeostasis. Misbalance and the resulting involvement of MMPs in disease, however, have been shown for a number of pathologies spanning from cancer to cardiovascular diseases and to neurodegeneration (Sbardella et al., 2012). The protective effect of clarithromycin in the case of autoimmune myocarditis appears to be implemented through the inhibition of the MMP-9 activity (Hishikari et al., 2010). In the long run, however, a short-term clarithromycin administration in patients with coronary heart disease for clearance of suspected infections results in increased risk of mortality (Gluud et al., 2008).

Immunosuppressive activities of macrolides have been known for almost four decades now. The first macrolide with this activity, rapamycin (also called sirolimus), was discovered by Brazilian researchers during a screening program for antifungal compounds produced by soil bacteria (Vézina et al., 1975). But its use as an antifungal antibiotic has been abandoned due to potent immunosuppressive and antiproliferative activities. Its antiproliferative action is realized through the formation of an active complex with its cytosolic receptor protein, FKBP12, and targeting of a putative lipid kinase termed target of rapamycin (TOR; Brown et al., 1994; Sabers et al., 1995; Wiederrecht et al., 1995). The loss of TOR function leads to the inhibition of G1- to S-phase progression in various sensitive cells. The immunosuppressive activity of rapamycin is also realized via the same protein kinase inhibition pathway affecting cell-cycle proliferation of lymphoid cells (Abraham, 1998).

The TOR complexes regulate cell growth and metabolism in response to environmental and intracellular cues and are comprised of two distinct multiprotein complexes: TOR complex 1 (TORC1), which is sensitive to rapamycin, and TORC2, which is not (Wullschleger et al., 2006). Dysregulation of these complexes is associated with various pathologies, including cancer, cardiovascular diseases, autoimmunity, metabolic disorders, and neurodegenerative diseases. Thus rapamycin and its derivatives can be used for treatment of a variety of diseases (Cruzado, 2008). It is also potentially useful for treatment of substance abuse conditions, alcohol abuse in particular, since inhibition of TORC1 by rapamycin disrupts alcohol-associated memory reconsolidation, leading to a long-lasting suppression of relapse (Barak et al., 2013). Currently it is approved for prevention of transplant rejection, and its latest derivative, everolimus, is widely used to prevent the rejection of heart, lung, kidney, or liver allografts (Gurk-Turner et al., 2012). Since TOR complexes are evolutionary conserved and involved in very fundamental biological processes in the cell, pharmacological inhibition of TOR signaling by rapamycin increases the lifespan of yeasts and higher eukaryotes (Powers et al., 2006). The use of rapamycin in humans as an anti-aging agent is uncertain because of side effects; this use will require the development of safer derivatives, termed rapalogs (Lamming et al., 2013).

Another macrolide compound with a potent immunosuppressive activity was discovered in 1984 during the screening program of Fujisawa Pharmaceutical Company aimed at compounds that reduce the risk of transplant rejection (Kino et al., 1987). This 23-membered macrolide lactone, isolated from *Streptomyces tsukubaensis* and called FK-506 (later also called tacrolimus or fujimycin), was initially approved in 1994 for the prophylaxis of liver transplant rejection, and since then the range of its use has expanded dramatically. In fact, it is considered as a cornerstone of modern immunosuppressive therapy and is used to treat allograft rejections that are resistant to other immunosuppressants (Rath, 2013). The effect of this class of macrolide immunosuppressants is also based on targeting the evolutionary conserved signal transduction pathways but via a mechanism other than that of rapamycin. In particular, FK506/FKBP complex inhibits the cytosolic phosphatase calcineurin, a key enzyme regulating the translocation of cytosolic components of various nuclear factors into the nucleus. Thus the blocked translocation of the cytosolic component of the nuclear factor of activated T cells (NF-AT) leads to its inability to activate a number of genes necessary for the proliferation of T cell such as IL-2 as well as for B cell help such as IL-4 (Ho et al., 1996).

Besides the extensive use in prophylaxis of transplant rejection, tacrolimus has demonstrated its efficacy and safety for a number of other inflammatory conditions. It has been successfully used in patients with inflammatory bowel disease, in particular for the treatment of severe cases (Baumgart et al., 2006) and for the induction of remission in refractory disease (Baumgart et al., 2008). In dermatology, the success of this drug is due to its topical effectiveness while cyclosporine, a drug with a similar mechanism of action, is topically non-responsive (Mrowietz, 1992). In general, many macrolide immunosuppressants, due to their chemical structure, are highly efficient in the topical form and are used extensively to treat many dermatological disorders (Mrowietz, 1999). They lack the skin-thinning side effect of corticosteroids and, therefore, can be used for extended periods of time and in areas with thin skin.

Macrolides are among the safest antibiotics in clinical use, with very few severe side effects (Periti et al., 1993). The most frequent manifestation is gastrointestinal disturbance occurring in 15–20% of patients on erythromycins and in 5% or fewer patients treated with novel macrolides. Transient deafness and allergic reactions are highly unusual and usually associated with the older macrolides. Clarithromycin and erythromycin may potentiate calcium-channel blockers by inhibiting cytochrome P450 isoenzyme 3A4 (Wright et al., 2011). Therefore, the concomitant use of calcium-channel blockers and these antibiotics may result in significant hypotension and shock (Wright et al., 2011; Henneman and Thornby, 2012). Although the risk is small, it can greater among the elderly and patients with multiple comorbidities.

In a subset of patients the use of tacrolimus for the management of hematopoietic allogeneic stem cell or solid organ transplantation is associated with a rare complication, posterior reversible encephalopathy syndrome (PRES; Wong et al., 2003; Bartynski et al., 2008; Hodnett et al., 2009; Wu et al., 2010; Hammerstrom et al., 2013). The main manifestations of PRES include altered mental status, seizures, visual abnormalities, and high blood pressure. While it is not clear how to manage the central nervous system (CNS)-related side effects, PRES associated with high blood pressure should include adequate blood pressure control (Hammerstrom et al., 2013).

Substantial progress has been made in identification of mammalian cell targets of macrolides. As discussed above, there is an extensive variety of targets for macrolides in the human body, and the list continues to grow. For instance, the immunomodulatory activities of macrolides can be mediated via the inhibition of production of many proinflammatory cytokines, the formation of leukotriene B4 (a neutrophils attractant), the formation of adhesion molecules necessary for neutrophil migration, and the release of superoxide anion by neutrophils (Tamaoki et al., 2004). The ketolide antibiotic telithromycin exerts powerful immunomodulatory and anti-inflammatory effects through NF-κB inhibition and enhancement of inflammatory cell apoptosis (Leiva et al., 2008a, b).

The macrolide antibiotic-binding human p8 protein has been cloned and identified using the phage display library approach (Morimura et al., 2008). This is a nuclear DNA-binding protein, which is strongly activated in response to several stresses, and, on the basis of functional similarity to HMG-I/Y-like proteins, it has been suggested that p8 may be involved in transcription regulation (Encinar et al., 2001; Hoffmeister et al., 2002). It plays an important role in such a basic biological process as ontogeny and hence is involved in a variety of developmental processes, such as pancreatic development in rats (Mallo et al., 1997), temporal expression of the beta subunit of luteinizing hormone (LHB) during gonadotroph development in mice (Million Passe et al., 2008), and mediation of gene expression in the diapause-destined crustacean *Artemia franciscana *(Qiu and MacRae, 2007).

In pathologies, the p8 protein is crucial for tumor development (Vasseur et al., 2002), and it is also involved in stress responses imposed by inflammation, tissue damage, and remodeling. Thus the list of pathologies includes diseases with an inflammatory component, acute pancreatitis for instance (Mallo et al., 1997). In cardiac pathology, p8 is broadly involved in cellular events leading to cardiomyocyte hypertrophy and cardiac fibroblast MMPs production, both of which take place in heart failure (Goruppi et al., 2007). Since clarithromycin, erythromycin, and azithromycin inhibit the binding of recombinant p8 protein to double-stranded DNA (Morimura et al., 2008), the anti-inflammatory effect of macrolides discussed above may be explained, at least in part, by the down-regulation of transcription of genes involved in the proinflammatory network. Interestingly, the same inhibitory effect has been observed with a structurally unrelated antifungal antibiotic dechlorogriseofulvin (Morimura et al., 2008), suggesting a potential overlap in recognition of structurally different antibiotic ligands by a single human molecular target.

The important difference between the antibiotic therapy of “classical” infectious diseases and chronic conditions such as cystic CF is the duration of antibiotic treatment. With the exception of *Mycobacterium tuberculosis *and few other difficult-to-eradicate infections, the antibiotic treatment period for infectious diseases is relatively short, while the maintenance therapy for chronic conditions is a long-term and perhaps life-long endeavor. One of the consequences of the maintenance therapy might be the selection for, and maintenance of, antibiotic resistance genes. In recent clinical trials evaluating the efficiency of the long-term azithromycin and erythromycin maintenance treatments in patients with non-CF bronchiectasis, the level of macrolide resistance significantly increased, despite the subinhibitory concentrations used (Altenburg et al., 2013; Serisier et al., 2013). Due to the importance of other than antibacterial activities of macrolides as well as to reduce the possibility of antibiotic selection for resistance, efforts have been made to design macrolide molecules with better anti-inflammatory activities (Kobayashi et al., 2013). Another approach to lessen the resistance burden is the design of macrolides that have their antimicrobial activities completely abolished but have other activities retained. It appears that the antimicrobial and anti-inflammatory activities of macrolides are independent and can be separated, thus opening the possibility of designing macrolide-based anti-inflammatory drugs lacking antimicrobial activities (Bosnar et al., 2012).

Thus the recent macrolide development efforts have bifurcated into two directions that are focused on designing and modification of macrolides better suited either for the antimicrobial or non-antimicrobial use. Historically, antibiotics (macrolides included), as their name implies, have been selected primarily for their antimicrobial activities, while other activities such as anti-inflammatory went unnoticed for a long time. In a recent non-antimicrobial antibiotic development, a novel macrolide, solithromycin, has displayed the capability to inhibit NF-κB and demonstrated better anti-inflammatory activities *in vitro *compared to more conventional macrolides used in the clinic such as erythromycin, clarithromycin, azithromycin, and telithromycin (Kobayashi et al., 2013). A better anti-inflammatory profile of this macrolide makes it a good candidate for the management of chronic respiratory diseases. Another macrolide with a significantly diminished antibiotic activity, 2′-desoxy-9-(S)-erythromycylamine, prevents neutrophil elastase-induced mucus stasis and dehydration and, therefore, may be used for the management of CF and COPD theoretically without affecting antibiotic resistance profile (Tarran et al., 2013). Synthetic mimetics of actin-binding macrolides may provide a range of designer compounds to treat actin-associated diseases (Perrins et al., 2008). On the other hand, there are continuous efforts to modify the existing macrolides to contain pathogens that are becoming resistant to older macrolides. Recent developments, for example, have been based on the use of azalide scaffold (Ištuk, 2011; Sugimoto et al., 2012), which was originally implemented in 9-dihydro-9-deoxo-9a-methyl-9a-aza-9a-homoerythromycin A (azithromycin), the antibiotic with outstanding pharmacokinetic properties (Amsden, 2001; Mutak, 2007).

## TETRACYCLINES

Tetracycline family of antibiotics is one of the best-studied examples of non-antimicrobial effects of antibiotics on the host. Tetracyclines possess multiple and potent biological activities, and minocycline, the best exemplary compound of this class, displays anti-inflammatory, neuroprotective, anti-proteolytic, and anti-apoptotic properties as well as inhibits angiogenesis and metastatic growth (Garrido-Mesa et al., 2013). In addition, it displays antioxidant activity, inhibits several enzyme activities, and regulates immune cell activation and proliferation.

Similar to the macrolides discussed above, tetracyclines are able to inhibit MMPs; this discovery was actually made in 1983, i.e., before the discovery of the corresponding activity among macrolides (Golub et al., 1983). As mentioned before, MMPs are involved in a number of pathologies, including metastatic growth, cardiovascular diseases, neurodegeneration, and a variety of inflammatory conditions (Sbardella et al., 2012). Thus the inhibition of MMPs may be a valuable option in treatment of a broad spectrum of diseases. Interestingly, the inhibitory effect on MMPs is realized through several targets/mechanisms ranging from indirect effects of regulatory network to down-regulation of expression and to direct interference with enzymatic activity. These activities can include binding divalent cations such as Ca^2^^+^ and Zn^2^^+^, inhibition of neutrophil migration and degranulation, and suppression of synthesis of oxygen radicals (Gabler and Creamer, 1991). Administration of minocycline to diabetic rats, for example, normalized the activity of four MMPs, while in *in vitro *assays minocycline inhibited only collagenase and gelatinase activities, with no inhibition of elastase and β-glucuronidase (Chang et al., 1996). The inhibitory effect of tetracycline on stromelysin is mediated via transcriptional inhibition involving sequences upstream of the activating protein complex 1 binding site (Jonat et al., 1996). But doxycycline, for example, down-regulates MMP-8 induction at both the mRNA and protein levels (Hanemaaijer et al., 1997). It also disrupts the conformation of the hemopexin-like domain of MMP-13 and the catalytic domain of MMP-8 (Smith et al., 1999).

Presently the only MMPs-targeting tetracycline that has been approved by the US Food and Drug Administration (FDA) and other national regulatory agencies in Canada and Europe is the low-dose formulation of doxycycline for the adjunctive treatment of chronic periodontal disease (Gu et al., 2012). Other conditions for potential application of low-dose tetracyclines to inhibit the pathological effects of MMPs are: (i) cardiovascular diseases such as coronary artery disease (Payne et al., 2011), hypertension (Castro et al., 2011), atherosclerosis (Gu et al., 2011), and abdominal aortic aneurysm (Abdul-Hussien et al., 2009); (ii) pulmonary diseases such as acute respiratory distress syndrome (ARDS) for which there is no approved medication (Roy et al., 2011), and COPD (Dalvi et al., 2011); (iii)metastatic cancers (Lokeshwar, 2011; Richards et al., 2011); and (iv) systemic bone loss conditions (Payne and Golub, 2011).

In many studies tetracyclines have demonstrated excellent anti-inflammatory activities achieved through the inhibition of chemotaxis, granuloma formation, nitric oxide production, and protease activities (Weinberg, 2005; Webster and Del Rosso, 2007). Positive effects of minocycline have been observed in animal models of rheumatoid arthritis (RA; Sewell et al., 1996), and this effect has been confirmed in several clinical trials as well (Greenwald, 2011). Tetracycline treatment of RA, however, is not widespread because of the almost universal use of methotrexate. Based on the results of successful clinical trials, subinhibitory concentrations of doxycycline and minocycline were approved by the FDA for the treatment of skin conditions and infections that have a substantial inflammatory component (Del Rosso, 2007). Another FDA approval, after successful clinical trials, has been obtained for the long-term management of chronic periodontitis by subantimicrobial doses of doxycycline (SDD) (Caton and Ryan, 2011). This host modulatory therapy is directed against excessive MMPs activities that are implicated in degradation of connective tissue collagen surrounding and supporting the teeth. This effect may have broader implications for other bone loss conditions (Payne and Golub, 2011).

Following the first reports on the neuroprotective effects of minocycline in animal models of cerebral ischemic injury (Yrjanheikki et al., 1998, 1999), doxycycline was proposed as a candidate for clinical trials of acute neurologic injury (Elewa et al., 2006). Among other tetracyclines, the neuroprotective potential of the second-generation antibiotic, minocycline, is remarkable. Its effect has been confirmed in experimental models of ischaemia, traumatic brain injury and neuropathic pain, and of several neurodegenerative conditions including Parkinson’s disease (Wu et al., 2002), Huntington’s disease (Chen et al., 2000; Wang et al., 2003), Alzheimer’s disease (Choi et al., 2007), amyotrophic lateral sclerosis (ALS; Tikka et al., 2002; Zhu et al., 2002), multiple sclerosis (MS; Metz et al., 2004; Zabad et al., 2007), and spinal cord injury (Marchand et al., 2009).

Presently, the results of several clinical trials aimed at the estimation of neuroprotective effects of minocycline are available, although with rather discouraging outcomes compared to animal studies. A phase III randomized trial of minocycline in ALS patients actually demonstrated a harmful effect of minocycline (Gordon et al., 2007). A futility study of minocycline in Huntington’s disease precluded proceeding with a phase III clinical trial (Schwarz et al., 2010). A prospective study with a cohort of multiple-system-atrophy Parkinson-type patients failed to show a clinical effect of minocycline on severity of symptoms (Dodel et al., 2010). Although the results of a phase II placebo-controlled randomized trial of minocycline in acute spinal cord injury did not establish efficacy, several outcome measures had a tendency toward improvement (Casha et al., 2012).

Treatment of a psychiatric illness relies on a combination of psychological and biological approaches. The latter have been for a long time focused on pharmacological interventions targeting mainly the neurotransmitter systems, but there is a growing body of evidence that these conditions are system-wide and include oxidative stress, inflammation, changes in glutamatergic pathways and neurotrophins as well (Dean et al., 2012). Minocycline is known as a modulator of glutamate-induced excitotoxicity and, in addition, it possesses antioxidative, anti-inflammatory, and neuroprotective properties. Pleiotropic properties of minocycline targeting multiple proteins and cellular processes implicated in the pathoetiology of mood disorders make it a suitable candidate for treatment of depression (Soczynska et al., 2012). It may be a valuable adjunctive therapeutic agent to antipsychotic medication in patients with schizophrenia as well (Miyaoka, 2008). In a clinical ad-on trial, minocycline treatment of early-phase schizophrenia patients improved negative symptoms and cognitive functions (Levkovitz et al., 2010). A recent preliminary open-label study has suggested that minocycline, in combination with antidepressants, is effective and well-tolerated in the treatment of unipolar psychotic depression (Miyaoka et al., 2012). In a mouse model of Fragile X syndrome (FXS), an inherited disorder with intellectual disability and behavior at the extreme of the autistic spectrum, minocycline showed potential to treat mental retardation and associated behavior (Bilousova et al., 2009; Rotschafer et al., 2012). Recent FXS clinical trials have indicated that minocycline may be effective in treating human patients as well (Siller and Broadie, 2012). In a number of cognitive impairment models minocycline treatment demonstrated promising results (Jin et al., 2013; Kong et al., 2013; Li et al., 2013). In a recent clinical trial evaluating the effect of minocycline on HIV-associated cognitive impairment, however, no significant improvement in cognitive functions has been found (Nakasujja et al., 2013).

Microglia are glial cells, the only resident immune cells in the CNS that respond to infections and brain injury and are actively involved in brain development and function as well as in neurodegenerative disease (Miyamoto et al., 2013). In the normal brain, these cells contribute to neuronal proliferation and differentiation, pruning of dying neurons, synaptic remodeling, and clearance of debris and aberrant proteins (Harry, 2013). Analogous to the activities of immune cells, activated microglia release cytokines, chemokines, nitric oxide, and reactive oxygen species (Harry, 2013). Dysfunctions in the homeostatic role of microglia, however, can affect neuronal functions such as cognition, personality, and information processing (Miyamoto et al., 2013).

Minocycline is known as the only drug capable of inhibiting the activation and proliferation of microglia (Tikka et al., 2001). In pathologies such as global brain ischemia, suppression of microglial activation by tetracyclines has a neuroprotective effect with a much better survival rate of CA1 pyramidal neurons (Yrjanheikki et al., 1998). The neuroprotective effects of tetracyclines in brain hypoxia are mainly due to the selective down-regulation of proinflammatory cytokines and compounds in the microglia (Lai and Todd, 2006). Overly rapid correction of chronic hyponatremia can lead to a severe demyelination disease, and the inhibition of microglial activation by minocycline prevents neurologic impairment and improves the survival rate (Suzuki et al., 2010). Minocycline also protects against microglial activation, neuronal death, and cognitive impairment caused by severe hypoglycemia in diabetic patients (Won et al., 2012). In the normal brain, the corresponding activity of minocycline modulates human social behavior leading to a more situation-oriented decision-making, possibly by suppressing the effects of personality traits (Kato et al., 2012). Minocycline also significantly reduces the risky trusting behavior in human economic exchange (Watabe et al., 2013).

This, not exhaustive but still impressive, list of conditions and diseases that can be treated with tetracyclines suggests the pleiotropic effects and the presence of multiple targets and receptors for which tetracyclines are ligands. In addition to the discussed above, the known anti-inflammatory activities of minocycline are achieved through the inhibition of expression of nitric oxide synthases (Amin et al., 1996), suppression of B and T cell function (Sewell et al., 1996), reduction of cyclooxygenase-2 expression and prostaglandin E(2) production (Yrjanheikki et al., 1999), and up-regulation of IL-10 (Ledeboer et al., 2005). Anti-apoptotic effects are believed to be due to inhibition of activity of caspase-1 and caspase-3 (Chen et al., 2000), inhibition of the phosphorylation of p38 mitogen-activated protein kinase (MAPK) (Du et al., 2001; Joks and Durkin, 2011), and inhibition of mitochondrial permeability-transition-mediated cytochrome *c* release (Zhu et al., 2002). By virtue of its molecular structure, minocycline is also an effective antioxidant with a radical scavenging potency similar to vitamin E thus providing excellent protection against oxidative stress (Kraus et al., 2005).

Tetracyclines are generally well-tolerated but under particular environmental conditions and in a subset of certain age and disease cohorts may provoke adverse reactions. One of the most known and well-studied side effects of tetracycline administration are cutaneous adverse events due to the increased photosensitivity of the skin, typically to the UVA spectrum of light (Drucker and Rosen, 2011; Glatz and Hofbauer, 2012). Clinically these effects can be divided into two groups, phototoxic and photoallergic reactions. The former is due to the formation of reactive oxygen species, with the impairment of many cellular macromolecules, thus leading to inflammation and apoptosis, while the latter is a type IV hypersensitivity reaction resembling eczema. The preventive measures include avoiding direct sunlight and the use of sunscreens. The culprit drug can be withdrawn if the reactions persist (Glatz and Hofbauer, 2012).

Tetracyclines are generally not recommended for pediatric patients because these compounds chelate calcium ions, which are incorporated into teeth, resulting in discoloration of both the primary and permanent dentitions (Sánchez et al., 2004). There are many case reports on the association of tooth, bone, nail, and scleral pigmentation following minocycline administration in adults as well but no systematic studies have been done in this area.

Many inflammatory diseases such as Alzheimer’s, Parkinson’s, Huntington’s, familial Mediterranean fever, and others tend to display a substantial protein deposition bias, where a normally soluble protein is deposited in an insoluble amyloid form (Carrell and Lomas, 1997). The deposits interfere with cellular functions, eventually leading to the cell death (Thomas et al., 1995). Tetracyclines are known to inhibit the deposition process (Sirangelo and Irace, 2010) but at the cost, by keeping the amyloid protein in a pre-fibrillar, highly cytotoxic state (Malmo et al., 2006). So care should be exercised to avoid the toxic effects of oligomeric species during tetracycline therapy.

In a development analogous to the search for non-antimicrobial macrolides there have been a series of works aimed at designing tetracycline derivatives with diminished antimicrobial activities while retaining or enhancing other activities important for their non-antimicrobial use. It needs to be noted here that the works with non-antimicrobial tetracycline derivatives have been actually initiated earlier than those with macrolides, i.e., shortly after the discovery of the host modulating properties of low-dose tetracyclines 30 years ago (Golub et al., 1983). However, a closer look into literature shows that the host modulating effects of antibiotic derivatives were detected even earlier, in 1950 (Stokstad and Jukes, 1950). In one of their experiments, the discoverers of growth-promoting antibiotics found that the cultural supernatant of *S. aureofaciens*, in which the antibiotic activity of aureomycin is destroyed by alkaline hydrolysis, still enhanced the growth and improved survival of chicks when added to feed (Stokstad and Jukes, 1950). Thus, the loss of antimicrobial activity of aureomycin has not compromised its other biological activities such as growth promotion. It is unfortunate that this interesting observation has been left without attention it deserves for so many years.

Chemically modified tetracyclines with no antimicrobial activity may have numerous applications without the associated risk of selecting for antibiotic resistance. For example, chemical conversion of tetracycline hydrochloride to the analog with no antimicrobial activity, de-dimethylaminotetracycline, has not compromised the collagenase inhibitory activity of the original molecule (Golub et al., 1987), while not affecting the tetracycline resistance profiles of gut and oral microbiota (Golub et al., 1991). COL-3, a chemically modified tetracycline with a MMP inhibitor activity, has showed promising results in the treatment of AIDS-related Kaposi’s sarcoma (Dezube et al., 2006). Moreover, the use of modified tetracyclines has showed promising results in many fields, including ophthalmologic diseases (Federici, 2011), dentistry (Grenier et al., 2002; Gu et al., 2012), cardiovascular pathologies (Salo et al., 2006; Gu et al., 2011), various types of cancer (Lokeshwar, 1999, 2011; Syed et al., 2004; Zhao et al., 2013a), and other conditions with excessive MMPs activities (Golub, 2011).

Another direction in the development of tetracyclines is understandably focused on the design of drugs with better antimicrobial activities and pharmacokinetic properties. Despite being highly efficient upon introduction in the clinical practice in the 1950s, the widespread resistance to the first- and second-generation tetracyclines made them essentially useless for treatment of many serious infectious diseases. One of the successful drug discovery programs resulted in a third-generation tetracycline called tigecycline (the minocycline derivative 9-tert-butyl-glycylamido-minocycline). The antibiotic is highly efficient against a broad range of pathogenic bacteria, including those resistant to the first- and second-generation tetracyclines (Bertrand and Dowzicky, 2012). Although it is on the list of reserve drugs, its use is steadily increasing (Huttner et al., 2012). Regrettably, similar to the fate of other antibiotics, the efficiency of tigecycline may start to deteriorate due to the penetration of tigecycline resistance into pathogenic microbiota (Aminov, 2013).

## β-LACTAMS

One of the most remarkable breakthroughs in the search for the therapy of neurodegenerative diseases has identified β-lactams as a very promising group of drugs. In a large screening effort involving 1,040 FDA-approved drugs and nutritionals, it has been discovered that the only drugs capable of regulating the expression and modulating the activity of the glutamate transporter subtype 1 (GLT-1) are β-lactams (Rothstein et al., 2005). Glutamate is a principal excitatory neurotransmitter in the CNS and contributes to learning and memory (Shigeri et al., 2004). The concentration of glutamate is mainly handled by GLT-1 (excitatory amino-acid transporter 2, EAAT2, responsible for 90% of glutamate uptake; Danbolt, 2001). Impairment of EAAT2 function leads to excess of glutamate and associated glutamate excitotoxicity destroying neurons and leading to neurodegenerative diseases such as ALS, epilepsy, and others (Maragakis and Rothstein, 2001). No practical pharmaceuticals modulating EAAT2 expression and activity were known until the discovery of such activity among β-lactams (Rothstein et al., 2005). A multi-phase randomized trial of ceftriaxone for treatment of ALS has been recently finalized (Berry et al., 2013).

Similar to other antibiotics, β-lactams can target many components of the eukaryotic cellular machinery, and the effects of β-lactams are not limited solely to the modulation of expression and activity of EAAT2. In a recent investigation of ceftriaxone as a potential therapy using a murine model of spinal muscular atrophy, the effects, in addition to the increase of EAAT2, also included the increase of the nuclear factor (erythroid-derived 2)-like 2, Nrf2, and the spinal muscular atrophy protein SMN (Nizzardo et al., 2011). The treatment resulted in significant amelioration of the neuromuscular phenotype and increased survival consistent with the protection of neuromuscular units through the activation of antioxidant response pathway governed by Nrf2. Another work has pointed to this target of ceftriaxone as well: together with the induction of the cystine/glutamate transporter SLC7A11 (formerly xCT), the neuroprotective effect of ceftriaxone *in vitro* is combined with the induction of Nrf2 consistent with the activation of the antioxidant defense system of the cell (Lewerenz et al., 2009).

In various models of brain injury ceftriaxone demonstrates strong neuroprotective effects, mainly via the up-regulation of GLT-1. In an experimental model of focal cerebral ischemia, the administration of ceftriaxone induces ischemic tolerance resulting in a better functional recovery of animals (Chu et al., 2007). A dramatic survival improvement can be seen in a rat model of stroke, if the animals are treated by a single injection of ceftriaxone 90 min after the middle cerebral artery occlusion (Thöne-Reineke et al., 2008). Pre-treatment with ceftriaxone also confers a significant neuroprotection in a cerebral ischemia/reperfusion injury (Verma et al., 2010). In a neonatal rat model of hypoxic–ischemic encephalopathy, pre-treatment with the antibiotic significantly reduces the brain injury scores and apoptotic cells in the hippocampus, restores myelination in the external capsule, and improves the posttraumatic learning and memory deficits (Lai et al., 2011). The neuroprotective effects of ceftriaxone are realized not only through the regulation of expression and activity of GLT-1, SLC7A11, and Nrf2. In a rat model of traumatic brain injury the antibiotic also significantly reduces the level of proinflammatory cytokines (Wei et al., 2012). Thus the improvement of cognitive functions and mitigation of brain edema after a brain injury, which is treated by posttraumatic administration of ceftriaxone, is a combined effect of reduced excitotoxicity and suppressed inflammation.

The potent immunomodulatory properties of β-lactam antibiotics, including inflammation control, are not limited to the sole example given above (Wei et al., 2012). In a mouse model of MS, ceftriaxone treatment indirectly hampered T cell proliferation and secretion of proinflammatory cytokines thus attenuating the disease course and its severity in this model of autoimmune CNS inflammation (Melzer et al., 2008). Interestingly, ceftriaxone has had no impact on the EAAT2 protein expression levels in several brain areas as well as on the glutamate uptake rate suggesting that in this model the positive effects of the antibiotic are not mediated through the modulation of glutamate concentration. Moreover, it seems that even the individual antibiotics within the β-lactam group may display differential immunomodulatory properties (Mor and Cohen, 2013). This mechanism operates via covalent binding of various β-lactams to cellular albumin and subsequent modulation of T cell function and gene expression.

β-lactams may be considered as valuable candidates for treatment of alcohol and other drug dependencies due to the capability of normalizing glutamate transmission, which is affected in addiction (Kalivas et al., 2009). To start with the simplest model: in planarians, ceftriaxone attenuates both the development of physical dependence and abstinence-induced withdrawal from cocaine, amphetamine, methamphetamine, and benzodiazepine (Rawls et al., 2008). In rats, the administration of ceftriaxone may suppress cue- and cocaine-induced relapses to cocaine-seeking behavior via up-regulation of GLT-1 and SLC7A11 (Sari et al., 2009; Knackstedt et al., 2010). Ceftriaxone also precludes cocaine sensitization and provides a long-term attenuation of cue- and cocaine-primed reinstatement of cocaine-seeking behavior, even after the cessation of antibiotic administration (Sondheimer and Knackstedt, 2011). In general, the antibiotic normalizes many aspects of glutamate homeostasis disrupted by the use of cocaine and, therefore, has the potential to lessen relapse episodes in human cocaine addicts (Trantham-Davidson et al., 2012). Currently there is no approved medication for the treatment of cocaine addiction, and the promising results obtained in the animal model experiments described above suggest that ceftriaxone (and possibly other β-lactams) can be considered as good candidates for clinical trials.

The biggest substance abuse problem is associated with excessive ethanol consumption, and β-lactams hold the potential to contribute to this problem as well. The biochemistry of ethanol addiction is more complicated compared to other drug dependencies, but one of its components discussed above, i.e., changes in glutamate transmission, is affected analogous to other drug addictions (Rao and Sari, 2012). Possibly the same mechanism of normalization, through the activation of GLT-1 by ceftriaxone, contributes to reduced ethanol consumption as well as to attenuation of relapse-like ethanol-drinking behavior in male alcohol-preferring rats (Sari et al., 2011; Qrunfleh et al., 2013). Reduction in the acquisition and maintenance of ethanol-drinking habit by ceftriaxone has also been verified for adolescent and adult female alcohol-preferring rats (Sari et al., 2013).

Enhanced glutamatergic transmission is a primary mediator of opiate dependence, and the counteractive effect of ceftriaxone prevents the development of morphine physical dependence in rats (Rawls et al., 2010a). Through the same remedial mechanism, the antibiotic reduces morphine analgesic tolerance (Rawls et al., 2010b) and suppresses opioid-induced hyperalgesia (Chen et al., 2012). The efficacy of ceftriaxone against drug dependence-related behavior extends to amphetamine (Rasmussen et al., 2011) and nicotine (Alajaji et al., 2013), as well as to the prevention of cannabinoid tolerance (Gunduz et al., 2011).

In general even the higher doses of ceftriaxone are tolerated well but care should be taken in neonates, especially those with hyperbilirubinemia and those receiving intravenous calcium solutions (Monte et al., 2008). There is no support for such restrictions in patients >28 days old (Steadman et al., 2010). The adverse reactions to ceftriaxone are caused by rapid intravenous injection, unlabeled use, and past history of allergic reactions to cephalosporins or penicillins (Shalviri et al., 2012). These risk factors can be easily managed under the normal clinical settings.

The host modulating properties of β-lactams have been discovered recently, and there is yet no data regarding the development of β-lactams with abolished antimicrobial activity. Unlike the macrolides and tetracyclines, the non-antimicrobial effects of β-lactams have been studied mostly with a single representative, ceftriaxone, and it is not clear if other representatives of this class of drugs possess similar properties. Another problematic area in ceftriaxone application for non-antimicrobial purposes is the concentrations used. The concentrations may be even higher than those for infectious disease treatment thus exerting potent selective pressure on commensal and pathogenic microbiota. This third-generation cephalosporin still remains a valuable therapeutic option for treatment of pneumonia, bacterial meningitis, Lyme disease, typhoid fever, and gonorrhea. Although the drug is losing its position as the last remaining option for first-line empiric treatment of, for example, *Neisseria gonorrhoeae *(Unemo and Nicholas, 2012), there are still some pathogens that have not acquired the corresponding resistance as yet. In this situation, exerting additional selective pressure by the non-antimicrobial use of ceftriaxone may complicate the management of a number of infectious diseases.

## DISCUSSION

Based on many promising results of the non-antimicrobial use of antimicrobials, the use of antibiotics for this purpose is expected to rise dramatically. Although being considered in the most recent clinical trials, a possible side effect of this therapy, such as the emergence and dissemination of antibiotic resistance among commensals and pathogens, has not received any considerable attention, and, in fact, the majority of clinical trials cited here have not monitored the occurrence of antibiotic resistance. There are some indications, however, that even low, non-selective concentrations of antibiotics used for a long time may affect the antibiotic resistance profile of human microbiota. For example, in a recent clinical trial of long-term, low-dose erythromycin on pulmonary exacerbations among patients with non-CF bronchiectasis, the proportion of macrolide-resistant oropharyngeal streptococci at the end of therapy has been substantially higher in the treatment group compared to the placebo group, 27.7 vs. 0.04%, respectively (Serisier et al., 2013). The use of azithromycin for the same purpose has resulted in the macrolide resistance rate of 88% compared to 26% in the placebo group (Altenburg et al., 2013). In this regard, the use of modified antibiotics with abolished antimicrobial activities may be helpful to circumvent such undesirable side effects of this type of therapy (Golub et al., 1991). Efforts to design newer antibiotic derivatives, which have no antimicrobial properties must be continued, especially for β-lactams that are used at the concentration range far exceeding those employed in infection control. For example, the recommended dose of ceftriaxone for treatment of gonorrhea has been increased from 125 to 250 mg due to the increasing resistance of *N. gonorrhoeae, *where for the majority of other infections the range rarely exceeds 1–2 g per day. But the most efficient management of, for example, ALS may require dosages up to 4 g/day (Berry et al., 2013). In combination with a generally long-term treatment required for this type of therapy (i.e., 20 weeks used in Berry et al., 2013), the total quantity of ceftriaxone consumed during a single course (560 g) might easily exceed the corresponding values used in a typical infection treatment by approximately 30–60 times. This may contribute to a significantly broader spread of resistance against third-generation cephalosporins among human commensals and, possibly, pathogens.

This clearly powerful selective pressure of ceftriaxone to be used for non-antimicrobial purposes brings forward another consideration for a more careful assessment of the biotic effects of antibiotics: that is, whether the effects of antibiotics are exerted solely via the human receptor-antibiotic ligand mechanisms. While the *in vitro *models largely have no issues with the influence of an indirect factor such as commensal microbiota, the corresponding experiments with animal models or human volunteers may need more careful interpretation because of a possible interfering effect of this often neglected variable. Our views on the role of commensal microbiota in human health and disease have undergone cardinal changes during the last decade from viewing it as a fairly passive bystander with a limited contribution to the host nutrition to an active organ of our body involved in many aspects of our metabolism, physiology, immunity, and disease. The field of host–microbiota research is overloaded with many works revealing the role of commensal microbiota in various pathologies ranging from inflammatory disorders to metabolic syndrome and to autism. It is, however, not possible to extend the frames of this review to include this fascinating area as well.

It is interesting to note, though, that there is an overlap between the range of diseases that can be treated by the biotic action of antibiotics and the diseases that have a substantial commensal microbial component. In Parkinson’s disease subjects, for example, the integrity of the intestinal lining is compromised thus allowing translocation of proinflammatory bacteria and bacterial products leading to the formation of the pathological hallmark of the disease, i.e., Lewy bodies with alpha-synuclein protein (Forsyth et al., 2011). As discussed above, the neuroprotective effects of minocycline may include reduced mitochondrial calcium uptake, stabilized mitochondrial membranes, reduced release of apoptotic factors, up-regulation of the anti-apoptotic protein Bcl-2, direct scavenging for reactive oxygen species, and inhibition of MAPKs (Orsucci et al., 2009). On the other side, minocycline may affect and modulate the microbiota, both commensal and translocated, thus reducing the load of proinflammatory bacteria and bacterial products. The effect on microbiota may be particularly profound for ceftriaxone, the biotic use concentration of which is much higher than typical antimicrobial concentrations used for infectious diseases. At concentrations used for typical antibacterial therapy, the ceftriaxone-induced dysbacteriosis may significantly change the fecal metabolome and affect the populations of T lymphocytes, their subpopulations in Peyer’s patches, and expression of various cytokines (Gao et al., 2012; Zhao et al., 2013b). Thus ceftriaxone considerably modifies the gut microbiome with the subsequent alteration of the host metabolome and immunity. It is currently unknown to what extent these secondary alterations contribute to the observed therapeutic effects of the non-antimicrobial ceftriaxone use in various diseases and pathologies. Because of the dual role of antibiotics, further research on biotic effects of antibiotics should incorporate both of these aspects in study designs.

Similarly, in the use of antibiotics as antimicrobial agents, their biotic effects are rarely considered as significant. Almost all infectious diseases, however, have a substantial inflammatory component, and, if left unchecked, the host’s proinflammatory responses may cause more harm than a pathogen would. In this regard, the anti-inflammatory activities of tetracyclines and macrolides discussed above may help to alleviate the overly aggressive inflammatory responses and prevent the resulting excessive tissue damage. In general, the number of diseases with a significant proinflammatory component, as exemplified above in this review, is on the rise, and one of the possible explanations for this phenomenon is offered by the hygiene hypothesis (Strachan, 1989). There used to be substantial exposure of humans to environmental bacteria before the advent of industrial food production resulting in essentially sterile food protected by preservatives, conservatives and freezing and generally higher hygienic standards of living. This exposure seems to have been an essential component of the immune system education and the lack of it results in an unbalanced immune system development, with a prominent proinflammatory bias. Thus antibiotics like tetracyclines and macrolides not only help to clear infections but also prevent generally excessive immune responses to infections characteristic for modern humans. Other antibiotics, however, may display the opposite biotic effects. For example, rifampin, a major drug used in tuberculosis treatment, increases inducible nitric oxide synthase expression and NF-κB activation and decreases PPARgamma expression (Yuhas et al., 2009), thus displaying strong proinflammatory properties.

The ligand and signaling activities of antibiotics that are beyond their intended antimicrobial use may explain the phenomena outside the human disease domain. Many are probably familiar with the growth-promoting effect of antibiotics on food animals. The growth-promoting antibiotics were completely phased out in the EU countries in 2006 because of their alleged contribution to the spread of antibiotic resistance, but it is still a legal practice in many countries. Despite the fact that the growth-promoting phenomenon was discovered more than 60 years ago, its mechanisms are still poorly understood. One of the possible explanations proposed has been the suppression of subclinical infections. But the concentrations of antibiotics used for growth promotion are well below the minimal inhibitory concentration (MICs) for the majority of pathogens. The irony of the situation is that it has been clear from the beginning that the growth-promoting effects of antibiotics are not due to antimicrobial activities. Since the very early days of the field it has been shown that even if the antibiotic activity of aureomycin in the culture supernatant of *S. aureofaciens *is destroyed by alkaline hydrolysis, it still retains the complete growth-promoting potential (Stokstad and Jukes, 1950). Thus Robert Stokstad and Thomas Jukes were the first to witness the biotic activity of the modified antibiotic but without realizing this. In the context of results discussed in this review, it is probable that the growth-promoting effect of antibiotics is largely due to the biotic signaling. This may affect the host and microbial components and can be realized through the modulation and pleiotropic regulation of the physiology and metabolic state of an entire microbiota as well as the regulatory mechanisms operating in the host animal. The regulatory effect of antibiotics on the microbial component has been discussed elsewhere (Davies et al., 2006; Linares et al., 2006; Yim et al., 2006, 2007; Martínez, 2008; Aminov, 2009; Romero et al., 2011). Other regulatory effects can be instigated through signaling to the host cells as has been demonstrated in this review. This could be modulation of immunity by suppressing subclinical inflammatory processes, which drain the host resources. It is probably not a coincidence that the vast majority of growth-promoting antibiotics belong to macrolides and tetracyclines that exert potent anti-inflammatory activities.

And finally, moving from the applications to the fundamentals, it remains to be discussed whether this multiplicity of antibiotic targets in the human/animal body we have just seen is merely accidental. Our perception of antibiotics as instruments of warfare in microbial communities is mainly influenced by the extrapolation of antibiotic use in clinical and veterinary microbiology, where high concentrations of antibiotics are used to eradicate bacterial infections in humans and animals. Several lines of evidence gathered in recent years suggest that antibiotic concentrations occurring in natural ecosystems are too low to kill the neighbors and, instead, they may play signaling and regulatory roles in microbial communities (Davies et al., 2006; Linares et al., 2006; Yim et al., 2006, 2007; Martínez, 2008; Aminov, 2009; Romero et al., 2011). The concept of antibiotics serving as signaling molecules in microbial ecosystems has gained considerable attention also in the context that it may explain the effect of low-dose antibiotics on the expression of genes regulating virulence, colonization, motility, stress response, biofilm formation, gene transfer, secondary metabolite production, and many other functions. If the sole role of antibiotics were limited to killing, then no effect of antibiotics would be seen below the MIC levels, and the effect would be neutral. However, it is not the case.

Are eukaryotes involved in this microbial signaling network? Before the advent of the multicellular organization about 0.5 billion years ago, the single-celled eukaryotes were probably well-incorporated into the then-existing microbial ecosystems, with the corresponding ancient signaling systems, and were also producing signaling substances themselves. They probably retained this capability even after the shift to multicellular organization, as may be exemplified by some of the ancient eukaryotic organisms having survived until the present days such as sponges which produce plethora of biologically active compounds, including antibiotics (Burkholder and Ruetzler, 1969; Laport et al., 2009). Further developments in multicellular organization in the eukaryotic world have led to a certain degree of isolation from the environment and the invention of intra-organismal signaling by hormones, but interaction and cross-talk with microbiota has remained an integral part of the lifestyle of eukaryotes. As discussed above, the microbiota is involved in many aspects of our metabolism, physiology, immunity, and disease through signaling to the host, but this is indeed a cross-talk with the microbiota also listening to the host signals (Burton et al., 2002; Sperandio et al., 2003; Clarke et al., 2006; Karavolos et al., 2008, 2013).

The importance of this host–microbe communication has been exemplified above by the hygiene hypothesis that explains a dramatic increase in the number of allergies and asthma in more developed countries due to limited exposure to environmental microbiota in the modern world. Another good example of the malfunctioning host–microbiota communication is the periodontal disease that affects 10–15% of adult population globally (Petersen and Ogawa, 2012). Although many suspected pathogens have been implicated in this disease, the underlying cause is more fundamental and mainly owes to the disrupted host–microbe interaction (Armitage, 2013; Bartold and Van Dyke, 2013). This disruption is the result of drastic changes in the lifestyle and diet imposed by the conversion to farming ca. 10,000 years ago and especially by the industrial revolution beginning from the 18th century. These changes shifted the oral microbial community to a disease-associated configuration (Adler et al., 2013), thus provoking the host to overly aggressive immune responses that result in tissue damage and disease progression. As discussed above, the situation can be somewhat corrected by the use of low-dose tetracyclines and tetracycline derivatives that suppress these responses, but this is a temporal solution which does not tackle the fundamental issue of the disease. The way of solving this is through reinstatement of oral microbiota from a less stable and diverse state, which would allow restituting the proper host–microbiota communication that has been selected and fine-tuned during prior long-term co-evolution of the host and microbiota.

Antibiotic signaling network is possibly one of the most ancient forms of inter-domain communication. It is not a coincidence that there are so many distinct molecular targets and receptors in eukaryotic cells for which antibiotics serve as ligands. Although therapeutic potential of this molecular cross-talk is well-understood and proven in certain pathologies and diseases, the effect on healthy individuals needs further elaboration. This is particularly important because the current exposure of humans to antibiotics, even in generally healthy populations, could be substantial but the consequences of this are poorly understood. For example, the phenomenon of the accelerated physiological development and tendency to be overweight in modern humans: is it the same effect of antibiotics similar to the growth promotion in food animals? The existing regulations require that the level of antibiotics for instance in meat products entering the human food chain should be below a certain tolerance level. But how the tolerance levels are defined? For example, detection of antibiotics and sulfonamides in bob veal calf carcasses are performed with the calf antibiotic and sulfonamide test (CAST) where *Bacillus megaterium* ATCC 9885 is used as the indicator organism (Dey et al., 2005). In order for a tissue sample to be considered safe, a zone of inhibition around a swab should be less than 18 mm. Under the identical incubation conditions antibiotic disks with 8.0 μg of sulfamethazine produce slightly smaller zones of inhibition, 17 mm in diameter (Dey et al., 2005). Moreover, the samples may contain the metabolized antibiotic residues that lost their antimicrobial activity but may still retain other biotic signaling properties as discussed in this review.

The use of antibiotics has been predominantly studied from the antimicrobial activity perspective and focused mostly on infection control and prevention of antibiotic resistance among pathogens. The advent and rapid expansion of non-antimicrobial application of antibiotics may enhance the already existing selective pressure of antibiotics and contribute to the present antibiotic resistance problem. Better understanding of biotic functions of antibiotics may help to design newer antibiotic derivatives with abolished antimicrobial activities and improved biotic functions.

## Conflict of Interest Statement

The author declares that the research was conducted in the absence of any commercial or financial relationships that could be construed as a potential conflict of interest.
